# Modeling Patient-Specific Magnetic Drug Targeting Within the Intracranial Vasculature

**DOI:** 10.3389/fphys.2018.00331

**Published:** 2018-04-19

**Authors:** Alexander Patronis, Robin A. Richardson, Sebastian Schmieschek, Brian J. N. Wylie, Rupert W. Nash, Peter V. Coveney

**Affiliations:** ^1^Centre for Computational Science, University College London, London, United Kingdom; ^2^Jülich Supercomputing Centre, Forschungszentrum Jülich, Jülich, Germany; ^3^Edinburgh Parallel Computing Centre, University of Edinburgh, Edinburgh, United Kingdom

**Keywords:** magnetic drug targeting, particle suspension, blood flow, lattice-Boltzmann method, multiscale, HemeLB

## Abstract

Drug targeting promises to substantially enhance future therapies, for example through the focussing of chemotherapeutic drugs at the site of a tumor, thus reducing the exposure of healthy tissue to unwanted damage. Promising work on the steering of medication in the human body employs magnetic fields acting on nanoparticles made of paramagnetic materials. We develop a computational tool to aid in the optimization of the physical parameters of these particles and the magnetic configuration, estimating the fraction of particles reaching a given target site in a large patient-specific vascular system for different physiological states (heart rate, cardiac output, etc.). We demonstrate the excellent computational performance of our model by its application to the simulation of paramagnetic-nanoparticle-laden flows in a circle of Willis geometry obtained from an MRI scan. The results suggest a strong dependence of the particle density at the target site on the strength of the magnetic forcing and the velocity of the background fluid flow.

## 1. Introduction

The accurate targeting of drugs toward specific regions of the human body promises to enhance future therapies and improve patient quality of life. The adverse effects of medications, such as those caused by chemotherapeutic drugs, may be minimized, while lower dosage requirements may decrease costs (Torchilin, 2000).

Drug targeting can be classified by the means as well as the level at which it is performed (Schleich et al., 2014). Viable mechanisms to enhance selective absorption include, but are not limited to, control of particle (drug carrier) size, addition of biochemical markers to drug carriers, and release of drug payloads within magnetized particles guided by external magnetic fields. Depending on the method employed, the term *drug target* may designate a certain type of tissue, specific cell type, or a location in space, such as the site of a tumor (Lockman et al., 2002).

Advances in technology have facilitated the production of micro- and nano-structures with great precision (Champion et al., 2007). In addition to the spherical particle carriers used in early experiments, state-of-the-art drug delivery systems incorporate bundles of nanotubes to encase biochemically active components. Such carrier structures can be designed to various specifications (Berry and Curtis, 2003; Tartaj et al., 2003), while a viable compromise between competing requirements may need to be found. For example, larger magnetic particles with micrometre radii are easier to manipulate via external fields, as the forces acting on them are proportional to their volume. On the other hand, the use of smaller particles (with dimensions of order tens of nanometres) has been found to enhance bioavailability and drug lifetime *in vivo* (Pankhurst et al., 2003; Nacev et al., 2012). Furthermore, the emergence of super-paramagnetic behavior, a finite-size effect that occurs for particle sizes below ~40 nm (Ulbrich et al., 2016), can substantially increase magnetic susceptibility, and hence enhance the response of particles to an external magnetic field. The use of such nanoparticles has received much attention in recent years, and the purpose of this paper is to report on the simulation of these, so as to inform on their design and aid future efforts.

The optimization of carriers and functionalization for drug targeting typically involves *in vivo* experiments and the immolation of animals. In this context, computational models can help to reduce the experimentation required. Within personalized medicine, the simulation, ahead of treatment, of magnetized particle suspensions in patient-specific geometries of vasculature derived from medical imaging data, would permit the selection of magnetic fields to control drug targeting.

There is significant interest in using magnetic drug targeting (MDT) for the treatment of diseases such as cancer (Tietze et al., 2012), due to the need to maximize damage to tumor cells (via the injection of highly toxic chemotherapeutic drugs) while keeping the exposure to healthy tissue in the remainder of a patient's body within tolerable levels. There have been several preclinical studies (Lübbe et al., 1996a; Goodwin et al., 1999; Alexiou et al., 2000), with a phase I clinical human trial carried out by Lübbe et al. using a single permanent magnet to concentrate epidoxorubicin-coated magnetic nanoparticles within shallow, inoperable tumors (Lübbe et al., 1996b, 2001), but with a number of issues identified (Shapiro et al., 2015). A major goal of MDT is to reach targets (e.g. tumors) deeper within the body, but different locations can require very different magnetic nanoparticle properties. *In vitro* experiments with flow phantoms can be used to determine the behavior of magnetic nanoparticles with different physiological and physical parameters (Radon et al., 2017). Simulation work by Nacev et al. suggests the use of a feedback control algorithm that modifies the applied magnetic field based on accurate real-time information on the distribution of particles (in principle obtainable from imaging) to focus the particles (on average) at a particular site (Nacev et al., 2012).

To be of most value in real world systems, MDT simulations must include a range of physical phenomena. Furthermore, so as to be able to resolve processes on relevant time and length scales, the simulation tools used must be computationally efficient. The ideal model would account for the mechanical properties of vessel walls, the complex rheological behavior of blood and its particulate nature, external magnetic fields and gravity etc. However, careful evaluation and control of the errors arising from different modeling assumptions and simplifications should enable reduced (and computationally efficient) models to be used with accuracy and reliability in clinical decision support. Moreover, multiscale models can inform coarse grained parametrization by quantifying effective parameter values.

There has been considerable development of models for MDT, focussing on the various scales and features of interest. Significant effort has been expended in modeling the MDT-relevant properties of the nanoparticle cores themselves (Winkler, 2017), e.g. through the use of the generalized finite element method (Plaks et al., 2003). The behavior of such nanoparticles in blood flow through simplified geometries has been explored using computational fluid dynamics (CFD) techniques such as the lattice-Boltzmann method (LBM) (in a simple channel) (Kandelousi and Ellahi, 2015), or the finite volume method (in a vessel bifurcation) (Larimi et al., 2014). Kenjereš and Righolt (2012) apply the conservation equations of mass and momentum (with an additional model describing a very dilute particle phase) for the simulation of blood flows carrying magnetic drug particles. Rukshin et al. modeled the motion of super-paramagnetic nanoparticles in a Poiseuille flow under the influence of an external magnet, taking into account the effects of Brownian motion and interactions with red blood cells, to determine particle arrival at the designated tumor site (found to depend dominantly on particle size, Rukshin et al., 2017).

In this work we aim to tackle comparatively much larger systems, with the exemplar case of a patient-specific vascular system (the circle of Willis) in a three-dimensional vascular system, concerning ourselves with determining the fraction of injected particles that reach a defined target site under varying physical parameters (of the nanoparticles) and physiological states (of the patient). We do not consider absorption into tissue at the target site, magnetically induced heating, biochemical reactions, or any other aspects specific to local treatment. Our strategy for the simulation of such a system relies on the LBM, which boasts extreme efficiency on massively parallel architectures, i.e. utilizing many compute units in an efficient manner (in section 4.2.1 we demonstrate strong scaling to approximately 100,000 cores). Through exploitation of its outstanding parallel performance, we use the LBM to reach a new level of understanding.

In this article we report on the integration of paramagnetic particles into HemeLB, an open-source lattice-Boltzmann code that is optimized for the large-scale simulation of sparse geometries on high performance computing resources (Mazzeo and Coveney, 2008). HemeLB is used for blood flow analysis (Bernabeu et al., 2013; Nash et al., 2014), and has been applied to gain insight into angiogenesis (Bernabeu et al., 2014) and vascular flow under different boundary conditions (Itani et al., 2015). Here, we assess the potential of HemeLB to evaluate magnetic drug targeting strategies in the context of personalized medicine. We develop, implement and validate a model for the simulation of magnetic particles in the circle of Willis, the central blood distribution system in the brain.

## 2. Materials and methods

### 2.1. Blood flow by the lattice-Boltzmann method

We simulate the flow of blood by the lattice-Boltzmann method (LBM), and assume incompressible flow at low Mach numbers. Our current approach approximates blood as a Newtonian fluid at a characteristic viscosity; for the systems presented herein, this provides a good approximation, and minimizes computational effort. Note that HemeLB allows for the simulation of non-Newtonian behavior, which may be used in conjunction with the particle model (Bernabeu et al., 2013).

The lattice-Boltzmann method describes fluid dynamics via a mesoscale approach. This replaces the single-particle distribution function *f*(***x***, ***c***, *t*) (at a position ***x***, continuous velocity ***c***, and time *t*) of the Boltzmann equation with a distribution function *f*_*i*_(***x***, *t*), where velocity space is reduced to a discrete set {***c***_*i*_}. After discretization in space and time, we have the lattice-Boltzmann equation (LBE),

(1) $$f_{i}(x+c_{i}\delta_{t},t+\delta_{t})-f_{i}(x,t)=-\Omega_{i}(f_{i}(x,t),f_{i}^{\text{ 0}}(x,t))+\delta_{t}F_{i}(x,t)$$

which describes the evolution of *f*_*i*_ by the streaming (left-hand terms) and collision terms. The last term in Equation (1) reproduces the effects of a hydrodynamic body force. Time is incremented by δ_*t*_ during each propagation step, and the discrete equilibrium distribution function *f*^0^ approximates the Maxwell-Boltzmann equilibrium distribution function to second order. The full derivation of the second-order accurate integration scheme for the forced LBE can be found in Nash et al. (2008).

Like the Bhatnagar-Gross-Krook (BGK) model of kinetic theory, the lattice Bhatnagar-Gross-Krook (LBGK) model describes particle collisions as a relaxation toward a local equilibrium, i.e.

(2) $$\Omega_{i}=\frac{1}{\tau }\left[f_{i}-f_{i}^{\text{ 0}}\right]$$

Herein, relaxation toward equilibrium on a single time scale τ is assumed. It can be shown that this approach approximates the Navier-Stokes equations (NSE) to second order (Qian and Orszag, 1993). For the purposes of this study, the LBGK collision model is used exclusively due to its simplicity.

#### 2.1.1. Parametrization and scaling

The lattice-Boltzmann method, as presented here, is athermal. The equation of state for a single fluid component, analogous to that of an ideal gas, relates the pressure to the lattice density ρ: $p=\rho c_{\text{s}}^{2}$. The lattice speed of sound *c*_s_ for D3Q19, the three-dimensional 19 velocity lattice, which is used throughout, is equal to $1/\sqrt{3}$. The simulation parameters δ_*x*_ (spatial discretization, i.e. the lattice spacing), δ_*t*_ (temporal discretization, i.e. the time-step length), and δ_*m*_ (the lattice mass) scale length, time and mass, respectively, such that the physical speed of sound is equal to *c*_s_δ_*x*_/δ_*t*_ and energy is non-dimensionalized by

(3) $$\delta_{m}\;\cdot \;\delta_{x}^{2}\;\cdot \;\delta_{t}^{-2}$$

Despite the athermal nature of the fluid model (by the LBM, which can be extended to give a thermal lattice-Boltzmann model), thermal energy *k*_B_*T* (where *k*_B_ is the Boltzmann constant and *T* is temperature) is considered in the calculation of a noise term, to be discussed in section 2.2, emulating the Brownian motion of particles (specifically, *k*_B_*T* appears in our calculation of particle diffusion by the Stokes-Einstein equation). True to the parametrization of blood flow we choose a temperature of 310.15 K or 37 °C.

To ensure consistent viscous behavior for a given set of scaling parameters, the dynamic viscosity

(4) μ=0.004 Pa s

and density of blood plasma

(5) $$\rho_{b}=1000\text{ kg }{\text{m}}^{-3}$$

are used to calculate relaxation parameters for the collision process. Note that, strictly speaking, μ is a function of the hematocrit (Pries et al., 1992). The lattice (kinematic) viscosity ν is related to the relaxation time τ by

(6) $$\nu =c_{\text{s}}^{2}\left(\tau -\frac{\delta_{t}}{2}\right)\text{ or},\text{in our case},\;\nu =\frac{1}{3}\left(\tau -\frac{1}{2}\right)$$

For numerical stability, the viscosity must be sufficiently large, i.e. τ > 0.5 (the limit of inviscid flow). In addition to this, the flow velocity must remain low relative to the speed of sound. We impose the Mach number limit $\text{Ma}=u/c_{\text{s}}^{2}<1/30$, corresponding to a maximum velocity of *u*_max_ ≈ 0.02 in lattice units.

### 2.2. Magnetized particles

Our strategy for the computationally-efficient simulation of paramagnetic particles suspended in blood combines an approach for the simulation of point-like particles (accounting for particle-fluid interaction) with a dipolar model. This pairing enables users of HemeLB, including clinicians and medical scientists, to study the efficacy of magnetic nanoparticles as a drug delivery system under the influence of an external magnetic field. We are particularly interested in understanding how such particles can be directed to problem sites, e.g. to the location of an inaccessible (by invasive procedures) tumor.

#### 2.2.1. Model for suspended particles

Our approach for the simulation of dilute suspensions, with particle sizes that are orders of magnitude smaller than the lattice spacing δ_*x*_, was developed with computational efficiency in mind; we aim to inform clinical decision-making, a time-critical process. The model is parameterized by particle radius *a*, position ***x***_*p*_ and velocity ***u***_*p*_. An efficient coupling mechanism is employed by neglecting particle inertia.

We list the source of forces that can be, by our implementation, applied to a paramagnetic particle (if, for a particular configuration, a forcing mechanism has a negligible impact on particle dynamics, it is deactivated to minimize computational effort): (1) a constant gravitational field; (2) hydrodynamic (Stokes') drag, due to the viscosity of the fluid (blood); (3) a (generally attractive) magnetic force due to paramagnetism; (4) a lubrication force, introduced to satisfy the wall-boundary condition on vessel walls and prevent the overlap of interacting particles; and (5) a stochastic force **F**_R_ (Brownian noise). For a paramagnetic particle under the action of these forces, we obtain (by balance of forces) the following for its motion:

(7) $$m{\dot{u}}_{p}=-6\pi \;\mu a[u_{p}-v(x_{p})]+F+F_{\text{R}}$$

where **F** is the combined sum of forces 1, 3, and 4 (excepting drag and **F**_R_), and ***v*** is the (interpolated) fluid velocity at the location ***x***_*p*_. By neglecting particle inertia, the left-hand side vanishes, and the hydrodynamic drag must balance the external forces on the particle. With the mobility β = 1/(6π μ*a*), the motion of a non-inertial particle in a dilute suspension can be expressed as

(8) $$u_{p}=v(x_{p})+\beta (F+F_{\text{R}})$$

which is dependent on the interpolated fluid velocity ***v***(*x*_*p*_) and the associated force terms. Note, in Equation (8), the effects of Brownian noise are only introduced through **F**_R_ – the fluid velocity ***v*** is deterministic. Noise is computed (by applying the fluctuation—dissipation theorem) to model the effects of Brownian motion.

In general, where the particle size is large relative to the lattice spacing δ_*x*_, a correction to the radius of the particle is required (Ladd, 1994; Nguyen and Ladd, 2002). Because we restrict our attention to the simulation of particles that are much smaller than the lattice spacing δ_*x*_ (the largest radius we consider is 0.5 μm with δ_*x*_ = 25 μm), we do not concern ourselves with the calculation of this correction. We similarly neglect the Faxén contributions in the particle equation of motion (Boivin et al., 1998; Horwitz and Mani, 2016), Equation (8) (discussed in section 5).

#### 2.2.2. Dipolar model

Since the calculation of inter-particle interactions can be costly, we exploit the dilute approximation and employ a simple dipolar model (DM) (Yung et al., 1998; Du and Biswal, 2014) to determine the (attractive) magnetic force between particles (dipoles) *i* and *j*, which we assume to be identical. The force on particle *i* due to particle *j* is

$$\begin{array}{l} F_{\text{M}}=\frac{3\mu_{0}}{4\pi \;r^{5}}[(m_{i}\;\cdot \;r_{ij})m_{j}+(m_{j}\;\cdot \;r_{ij})m_{i}+(m_{i}\;\cdot \;m_{j})r_{ij}\\ \;-5r^{-2}(m_{i}\;\cdot \;r_{ij})(m_{j}\;\cdot \;r_{ij})r_{ij}] \end{array}$$

where μ_0_ is the permeability, ***r***_*ij*_ is the connecting vector from *j* to *i*, and $m_{i}=4\pi a^{3}\chi_{v}\text{H}/3$ (and similarly for *j*). Note that we neglect variations in the magnetic field **H** over the size of a particle, and that χ_*v*_ is the effective volumetric susceptibility. We calculate **H** at the position of the interaction by Yung et al. (1998)

(9) $$H=\frac{1}{4\pi }\left[(m_{0}\;\cdot \;r_{0})\frac{3r_{0}}{r_{0}^{5}}-\frac{m_{0}}{r_{0}^{3}}\right]$$

where ***m***_0_ is the magnetic moment of a permanent magnet, and ***r***_0_ is the vector connecting the magnet and a particle. For the results presented in section 4, ***m***_0_ is imposed in the *x*-direction, i.e. perpendicular to the sagittal plane (see **Figure 2**). Equation (9) also gives the force exerted by the magnet on a particle.

**Figure 1:**
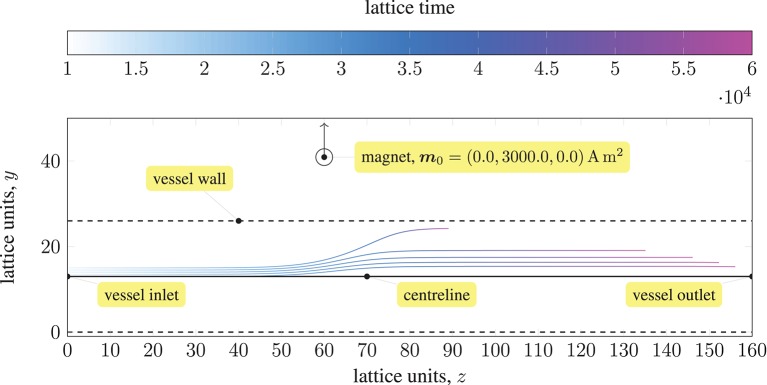
Trajectories of five paramagnetic nanoparticles (initially placed at the inlet of a three-dimensional Poiseuille flow) as they approach a permanent magnet that is external to the flow (represented by a circle). Deviation from the pressure-induced flow occurs once the magnetic attraction experienced by the particles is sufficiently large; the magnetic field is imposed in the *y*-direction (indicated by the arrow). The coloring of the trajectories represents the evolution of time. The force exerted by the magnet on each particle far exceeds that experienced between particles; hence, the particles do not converge.

We demonstrate the effects of this model by following the trajectories of 5 paramagnetic particles in a three-dimensional Poiseuille flow, as shown in Figure 1. A permanent magnet (on the *yz*-plane passing through the center of the vessel) is placed 0.0022 mm from the centerline. A magnetic moment of ***m***_0_ = {0.0, 3000.0, 0.0}A m^2^ is imposed. The pressure at the inlet (at *z* = 0) is 0.01 mmHg or 1.33 Pa, resulting in a pressure gradient of 103.9 Pa m^−1^. Initially, the evenly-spaced particles follow the pressure-induced flow, with the particle on the centerline at maximum (flow) velocity. As they approach the magnet, the particles experience a significant force that disrupts their motion; the particle closest to the magnet (i.e. the outermost) is significantly affected, with its streamwise velocity reduced such that it remains near to the wall of the vessel for a considerable time (relative to the other particles). Because the force exerted by the magnet on the particles is larger than the force experienced between particles (owing to paramagnetism), we do not see the trajectories of the particles converge. Note that to avoid divergence of the attractive forces, a lubrication force between particles is applied, ensuring that particles do not overlap.

#### 2.2.3. Lubrication forces

The wall-boundary interaction of particles is modeled by a lubrication force (ten Cate et al., 2002)

(10) $$F_{\text{L}}=6\pi \;\mu a^{2}(u_{p}\;\cdot \;{\hat{r}}_{w})\left[\frac{1}{h}-\frac{1}{h_{e}}\right]$$

with the particle-wall separation *h* = ||***r***_*w*_|| − *a* (***r***_*w*_ is the particle-to-wall vector), a cut-off distance *h*_*e*_ (for numerical efficiency, and dependent on the strength of interactions), and the velocity of the particle ***u***_*p*_. In ten Cate et al. (2002), the force from Equation (10) is compared to experimental data. In section 3.1, our implementation of the boundary condition is validated by comparison with the analytical predictions of Maude (1961).

The lubrication force between two identical particles is similarly given by Nguyen and Ladd (2002)

(11) $$F_{\text{L}}=\frac{6\pi }{4}\mu a^{2}(u_{ij}\;\cdot \;{\hat{r}}_{ij})\left[\frac{1}{h}-\frac{1}{h_{e}}\right]$$

with the relative velocity between particles ***u***_*ij*_ = ***u***_*i*_ − ***u***_*j*_, the separation between particles *h* = ||***r***_*ij*_|| − 2*a*, and a cut-off distance *h*_*e*_, which is not necessarily equal in value to that used for particle-wall lubrication.

### 2.3. Flow geometry

Acting as the central blood distribution system in the brain, the circle of Willis (coW) connects the inflow from the basilar and internal carotid arteries to the cerebral arteries via a circular system closed by communicating arteries. Studies have found considerable variation in the structure of this system (Kayembe et al., 1984; Eftekhar et al., 2006). Its inherent redundancy allows it to function despite the presence of deformed or missing subsystems.

**Figure 2:**
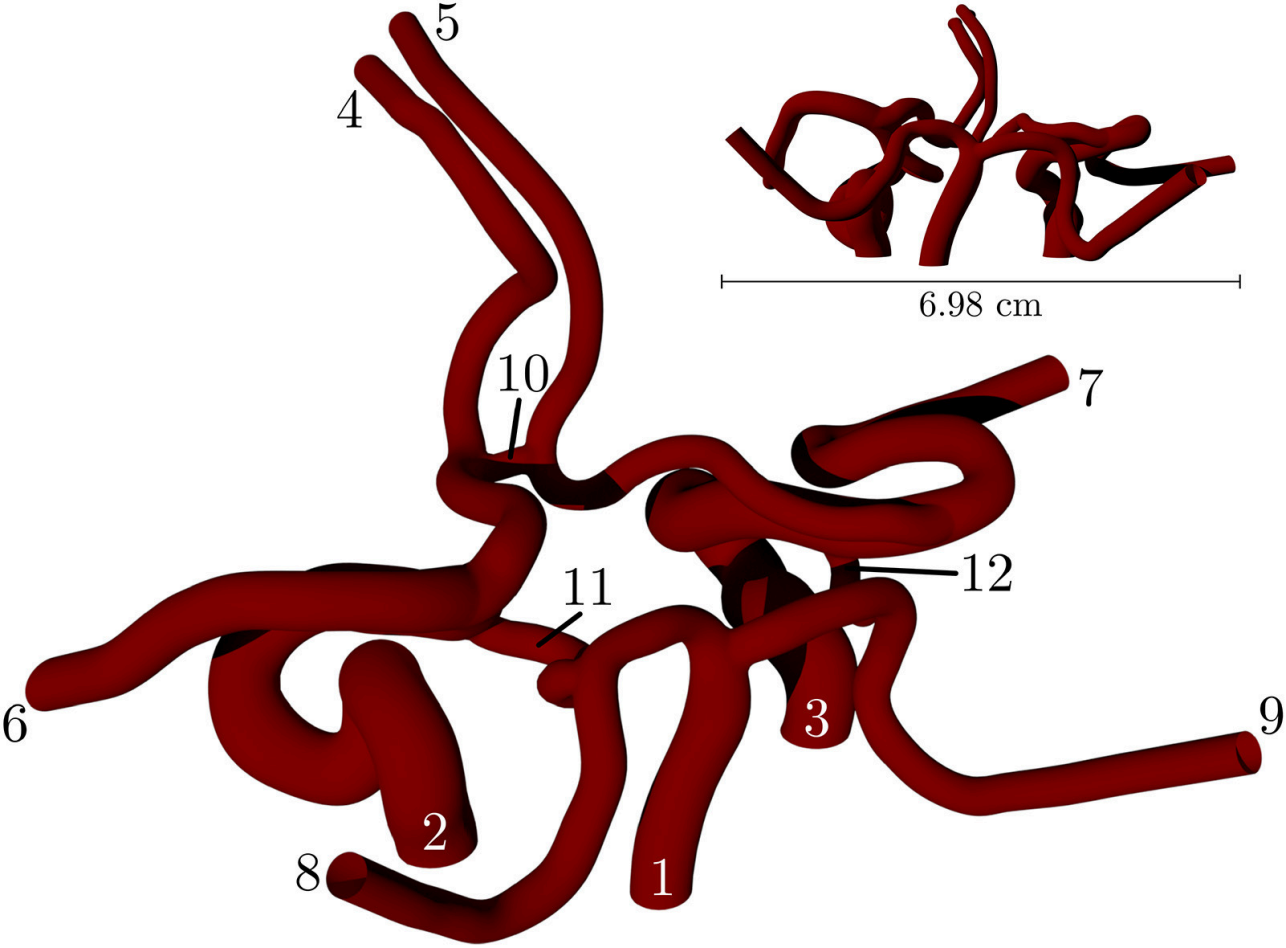
Volume rendering of the circle of Willis, constructed from an MRI scan of a human subject. The circle of Willis is the main blood distribution system in the brain, and is located roughly in the center of the head. The numbering of the inlet/outlet is to be cross-referenced with Table 1.

Figure 2 depicts a volume rendering of the structure of a complete coW (with lateral dimensions of order cm), obtained from a magnetic resonance imaging (MRI) scan. For details on the generation of this particular geometry, see Coogan et al. (2013). The geometry is used exclusively throughout, and is prepared for use by HemeLB using Palabos' (http://www.palabos.org) fully-parallelized voxelizer (indispensable when voxelizing large geometries with billions of lattice sites); our “common vascular pipeline” allows HemeLB and Palabos to share the same pre-processing workflow.

**Figure 3:**
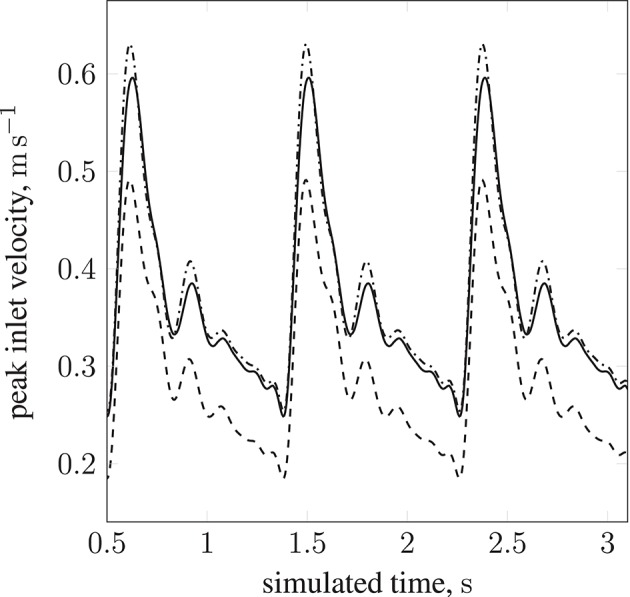
Peak inlet velocity for a resting patient (blood pressure: 80 mmHg, volumetric flow rate: 4.8 l min^−1^, heart rate: 68 bpm) in (1) the basilar artery (- - -), (2) the left internal carotid artery (—), and (3) the right internal carotid artery (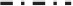). For each of the three inlets, the complete inlet-velocity profile is obtained by assigning weighting factors (of the peak velocity) to lattice sites that lie on the boundaries.

Table 1 lists the names of the modeled arteries with the boundary conditions employed. Boundary conditions at the inlet are approximated by a parabolic flow profile with a maximum flow speed informed by a 1D Navier-Stokes simulation (performed using PyNS, Manini et al., 2015) of the complete arterial network. The maximum velocity observed in the left internal carotid artery is *u*_max_ ≈ 0.63 m s^−1^ (see Figure 3). This value, in conjunction with the stability requirements introduced in section 2.1.1 and the spatial discretization δ_*x*_ = 25 μm (resulting in a simulation domain of 1.66 × 10^8^ lattice sites), leads to a time-step of 7.8 × 10^−7^ s. We use this lattice spacing (δ_*x*_ = 25 μm) throughout, with the exception of section 4.2.1, where we use δ_*x*_ = 15 μm to produce approximately 7.77 × 10^8^ lattice sites for our assessment of application scalability. Outlet boundary conditions assume a vanishing pressure gradient.

**Table 1:** The validation geometry is a magnetic resonance imaging (MRI) scan of the circle of Willis, with lateral dimensions of order cm.

**Index**	**Artery**	**Boundary condition**
1	Basilar	Neumann (inlet)
2	Internal carotid (left)	Neumann (inlet)
3	Internal carotid (right)	Neumann (inlet)
4	Anterior cerebral (left)	Dirichlet (outlet)
5	Anterior cerebral (right)	Dirichlet (outlet)
6	Middle cerebral (left)	Dirichlet (outlet)
7	Middle cerebral (right)	Dirichlet (outlet)
8	Posterior cerebral (left)	Dirichlet (outlet)
9	Posterior cerebral (right)	Dirichlet (outlet)
10	Anterior communicating	Not applicable
11	Posterior communicating (left)	Not applicable
12	Posterior communicating (right)	Not applicable

*The inlet boundaries 1, 2, and 3 (see text for details) are parameterized by 1D Navier-Stokes solutions for the full arterial network (Itani et al., 2015; Manini et al., 2015). At the outlet boundaries 4–9, a vanishing pressure gradient is enforced approximating constant pressure. The communicating arteries (10, 11, and 12) close the circular structure; no boundary conditions are applied to the limits of these arteries. The numbering of the inlet/outlet is to be cross-referenced with Figure 2, which shows the full circle of Willis*.

## 3. Implementation and validation

HemeLB is a lattice-Boltzmann implementation optimized for the simulation of sparse geometries by means of indirect addressing of lattice sites. The code is written in C++ and makes use of static polymorphism to allow the efficient selection of different lattice discretizations, collision models and boundary conditions. Parallelization is implemented via MPI. The HemeLB application relies on several external libraries for standardized tasks, such as XML processing, domain decomposition and unit testing (Groen et al., 2013). External tools are available for the creation of input files (including the previously mentioned voxelizer) and the post-processing and evaluation of extracted data. The code is open-source, licensed under the GNU Lesser Public License (LGPL), and is available at https://github.com/UCL/hemelb.

HemeLB supports D3Q15, D3Q19, and D3Q27 lattice discretizations, that is three dimensions comprising Q discrete lattice velocities; in this work we limit ourselves to D3Q19. Collision processes can be modeled either by the lattice Bhatnager-Gross-Krook (LBGK) scheme (as is the case in this work), relying on a single relaxation time, or by invoking a multi relaxation time (MRT) model. Furthermore a non-Newtonian approximation of a shear thinning fluid is available. The code supports various wall boundary conditions, including simple bounce-back, Guo-Zheng-Shi (Guo et al., 2002), Bouzidi-Firdaouss-Lallemand (BFL) (Bouzidi et al., 2001) (used exclusively, for its superior accuracy, in this work) and Junk and Yang (2005) (see Nash et al., 2014 for discussion of these).

**Figure 4:**
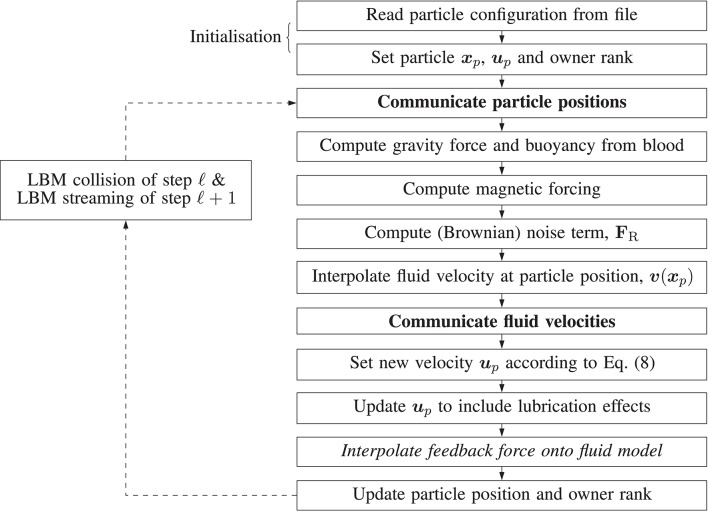
Illustration of the algorithm steps implementing the magnetic particle model in HemeLB. Arrows represent the progression of a time-step. Dashed arrows represent the progression of the time-step outside of the algorithm responsible for updating the paramagnetic particles, i.e. simulation is evolving according to the standard LBM procedure. Steps in boldface involve communication between processes. The italicized step is only performed if two-way coupling is enabled.

Figure 4 illustrates the algorithm which implements the paramagnetic particle model. After the LBM lattice velocity update, the particle update procedure begins. Firstly, particles are communicated between ranks; a particle is only communicated if (by the update of its position at the end of the previous step) it has moved to another rank, or its 3D Moore neighborhood spans multiple ranks (so that the interpolation of the fluid velocity can occur correctly; we refer to these as ghost particles). Once particles have been communicated, we zero the force on each and accumulate the new value as the sum of any external forces. As the fluid velocity is only calculated at lattice sites, interpolation is used to find ***v*** at ***x***_*p*_, as required by Equation (8). When mass and volume loading are sufficient (Birzer et al., 2012), the influence of the particles on the flow cannot be neglected. In this case, we enable two-way coupling and the forces exerted on the fluid by locally owned particles are then interpolated onto local lattice sites. The memory of particle momentum is carried by the fluid model, allowing the computational cost to be dramatically reduced (Ahlrichs and Dünweg, 1999; Nash et al., 2008).

### 3.1. Lubrication boundary condition

**Figure 5:**
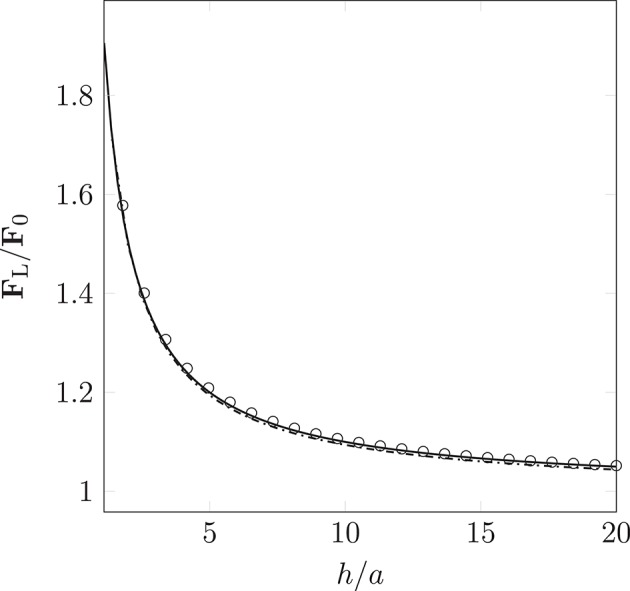
Non-dimensionalized lubrication force imposed by the lubrication boundary condition as a function of the particle's distance to the wall (the separation between the particle's surface and the wall). Measurements were taken for particle radius *a* = 25 nm (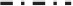) and 500 nm (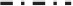). The simulation results approximate Equation (12) ° well. Deviations arise due to the discretization of movement within the LB time-step.

Wall-boundary conditions for the point-like particle model are implemented by introducing an additional force, Equation (10). We use a constant body force to drive monodisperse particles (of radii *a* = 25 nm and *a* = 500 nm) into a wall that is perpendicular to the instantaneous direction of motion. We record the resulting lubrication force experienced by each particle. Figure 5 shows the lubrication force imposed by the boundary condition as a function of the separation *h* (the distance of the particle to the wall). The measured lubrication force **F**_L_ and *h* are non-dimensionalized by the drag force **F**_0_ = 6π μ*a**u***_*p*_ and the particle radius *a*, respectively. A theoretical expression for the lubrication force,

(12) $$F_{\text{L}}=F_{0}\left(\frac{9}{8}\frac{a}{h}+1\right)$$

has been formulated by Maude (1961). For verification, we compare this to the simulated **F**_L_. As can be seen in Figure 5, the lubrication boundary condition approximates the theory well. The observed deviations are a result of the finite size of the simulation time-step, and the particle's non-continuous motion.

### 3.2. Inter-particle interactions in an external magnetic field

**Figure 6:**
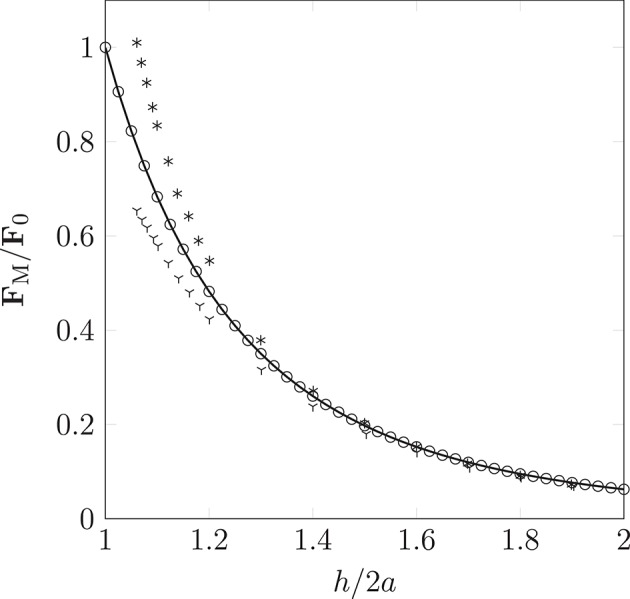
Non-dimensionalized forces acting on pairs of particles oriented parallel (according to theory 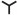 and the simulation 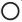) or orthogonal (according to theory * and the simulation 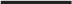) to a homogeneous magnetic field. Our simple dipolar model assumes the field is undisturbed by the inter-particle interaction. The validity of this simplification can be justified by considering the disparity in time scales of hydrodynamic and magnetic interactions (the latter can be assumed to occur instantaneously). As expected, the deviation caused by neglect of the rotational contribution is most pronounced as *h*/2*a* → 1, where *a* is the particle radius (of monodispersed particles), and *h* is the separation between interacting particles.

The dipolar model (DM) is evaluated by comparison of the simulated interaction force (obtained from Equation 9 as implemented in HemeLB) between two identical paramagnetic particles (oriented parallel and perpendicular to a constant external field) with solutions of the Laplace equation. Figure 6 clearly illustrates the isotropy of the approximation of the DM, which neglects contributions of the particle orientation. Note, the force **F**_M_ is normalized by the force encountered for touching particles of separation *h* = 2*a*; we refer to this maximum force as **F**_0_. As expected, the error increases as *h*/2*a* → 1. For separations exceeding *h* = 3*a* the approximation becomes more accurate, to within a few percent of the analytical solution. As *h* is increased further, we observe excellent agreement between the simulated result and theory. As our model requires the suspension to be dilute, the latter case, where *h* > 3*a*, will be most likely.

## 4. Results

In this section we present two simulations of paramagnetic particles suspended in blood while circulating in the circle of Willis: (1) a permanent magnet, assumed to be a pure dipole with ***m***_0_ = {3000, 0.0, 0.0}A m^2^, is held at a distance of 3 cm from the geometric center of the circle of Willis (shown in Figure 2), causing the particles to experience an attractive force that brings them together and toward the external magnet (source of the magnetization); (2) the magnet is removed and no attraction exists between any dipoles (paramagnetic particles). In both of these simulations, all other body forces listed in section 2.2.1 are active. The captured flow will first be presented, with illustrations revealing the behavior of particles through the coW, followed by an analysis of the computational performance of HemeLB when simulating such flows.

### 4.1. Simulations of paramagnetic particle suspensions

**Figure 7:**
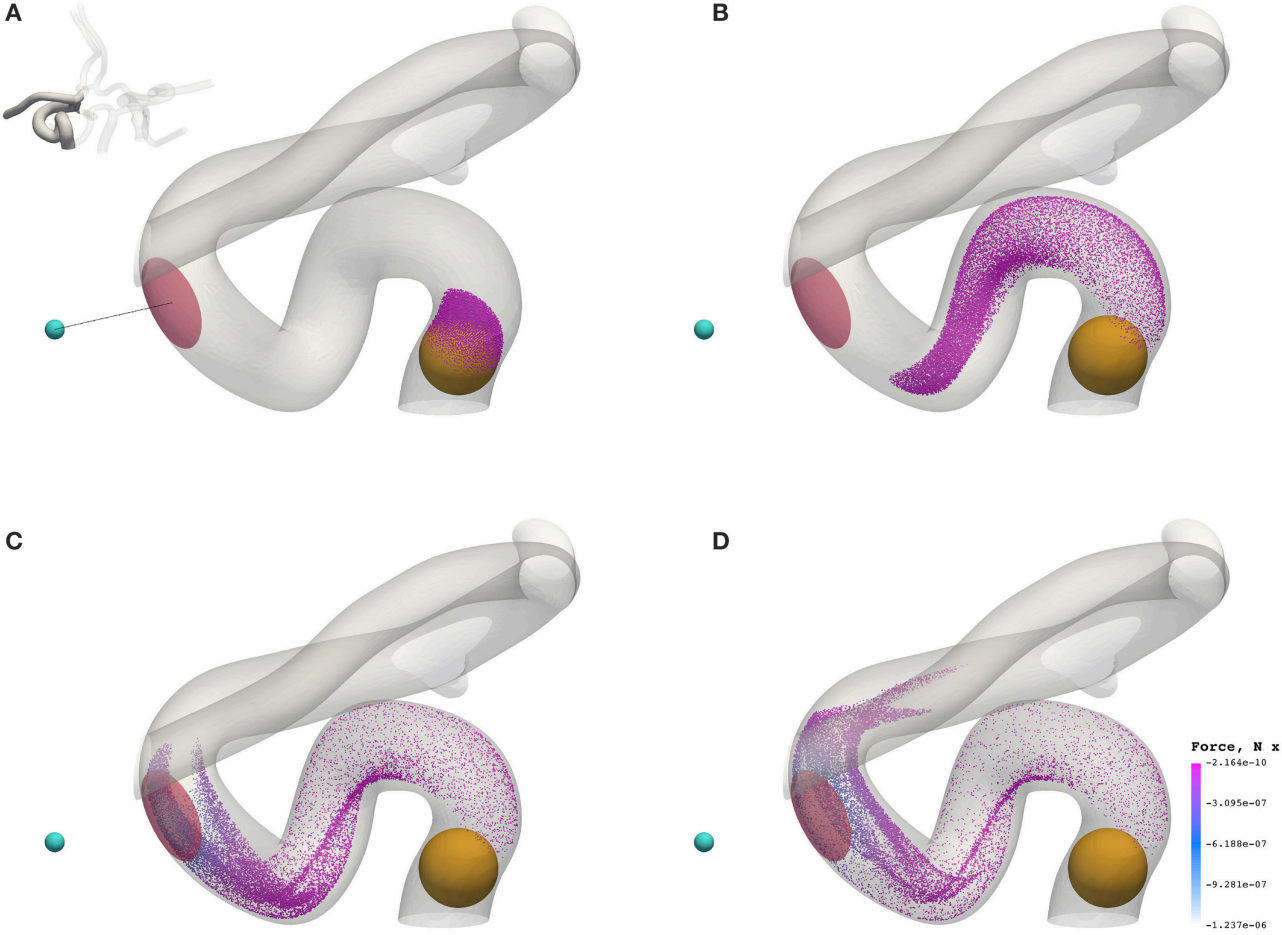
Particles (with radius *a* = 65 nm) traveling from the left internal carotid artery through the circle of Willis. Particles are colored by the magnetic force (in the *x*-direction) that they experience. The cyan sphere represents a permanent (and fixed) magnet. All particles are initially confined to the interior of the orange sphere, in which particle positions are randomly distributed. The pink volume (internal to the coW) represents some region of interest, e.g. a site requiring therapeutic attention, to which we force particles by virtue of the magnetic field; we record the instantaneous particle count in this region. **(A)** Particle positions at 0.078 s. **(B)** Particle positions at 0.273 s. **(C)** Particle positions at 0.351 s. **(D)** Particle positions at 0.39 s.

Figure 7 shows the transport of nanoparticles through the circle of Willis; initially, particle positions are randomly distributed (without overlap) within a sphere (colored orange in Figure 7, and shown only for illustrative purposes; it is not present in the simulation) at inlet 2 of Figure 2. Particles are colored by the *x* component of the magnetic force they experience as they travel. In Figure 7, the cyan sphere represents the permanent magnet that is responsible for the magnet field (with magnetic moment ***m***_0_ = {3000.0, 0.0, 0.0}A m^2^). The region of interest (RoI), colored pink, is a three-dimensional volume that we are attempting to target (e.g. the site of a tumor) using the nanoparticles. We simulate three cases, varying particle radius *a* (= 65, 105, and 500 nm) to study the efficacy of the magnet to direct the paramagnetic particles toward a site. Note that although particles are monodispersed (i.e. all of the same size) in all reported simulations, our method fully supports polydispersity (to be exploited in future studies). The visualizations shown here are for *a* = 65 nm, but particles are not shown to scale.

**Figure 8:**
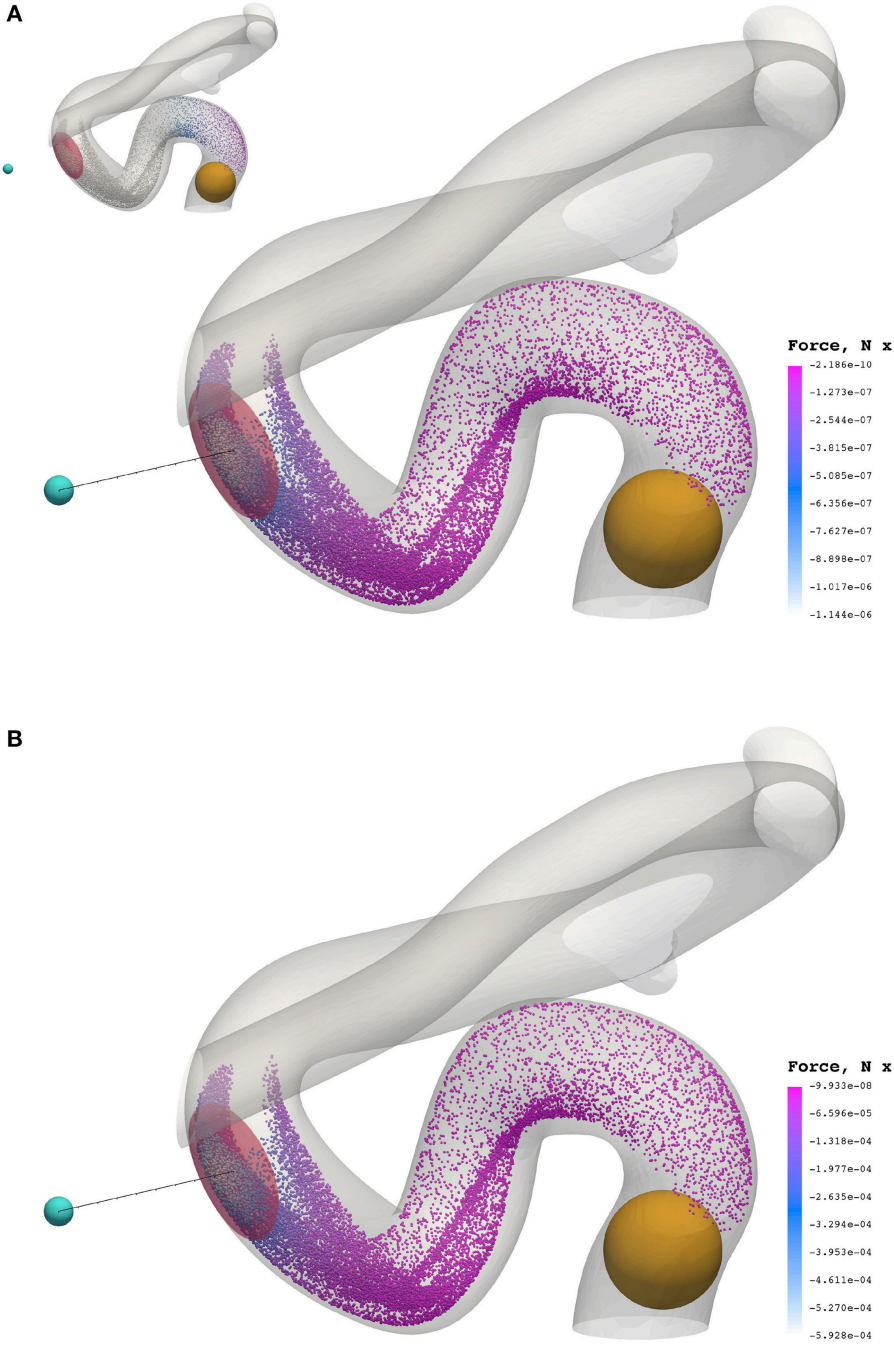
The *x* component of the magnetic force experienced by particles of radius *a* = 65 nm **(A)** and *a* = 500 nm **(B)** at 0.3549 s. To highlight the significant difference in force between cases, the inset figure in the top visualization applies the color scale limits for *a* = 65 nm on the case where *a* = 500 nm. The maximum force in the case of *a* = 65 nm is *F*_M_ = −1.144 × 10^−6^ N, whereas the maximum force in the case of *a* = 500 nm is *F*_M_ = −5.928 × 10^−4^ N.

Figure 8 presents a comparison of the magnetic force experienced by particles of radius *a* = 65 nm (top) and *a* = 500 nm (bottom) at 0.3549 s (smallest and largest radii considered). The maximum force in the case of *a* = 65 nm is **F**_M_ = −1.144 × 10^−6^ N, whereas the maximum force in the case of *a* = 500 nm is *F*_M_ = −5.928 × 10^−4^ N; two orders of magnitude separate the maximum force observed in these cases.

**Figure 9:**
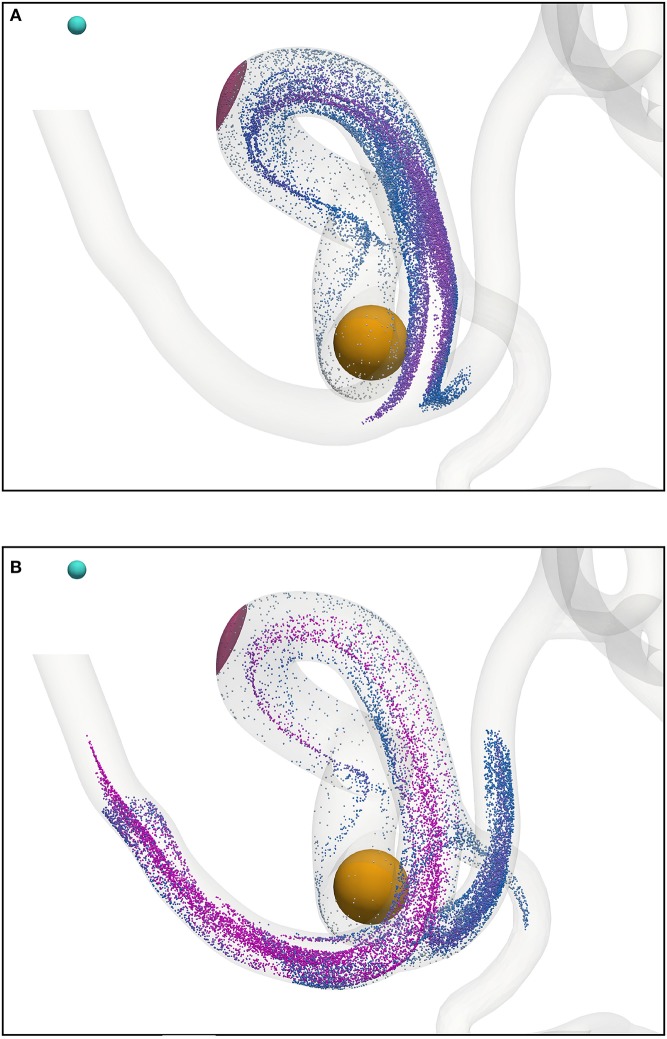
Particles (with radius *a* = 65 nm) traveling from the left internal carotid artery through the circle of Willis; particles leave through outlets 4, 6, and 8 (see Figure 2). Particles are colored by their velocity. The cyan sphere represents a permanent (and fixed) magnet. All particles are initially confined to the interior of the orange sphere, in which particle positions are randomly distributed. The pink volume (internal to the coW) represents some region of interest, e.g. a site requiring therapeutic attention, to which we force particles by virtue of the magnetic field; we record the instantaneous particle count in this region. **(A)** Particle positions at 0.468 s. **(B)** Particle positions at 0.546 s.

Beyond the small region shown in Figure 7, the particles continue to travel through the circle of Willis before exiting through the left anterior cerebral artery (outlet 4), the left middle cerebral artery (outlet 6), and the posterior cerebral artery (outlet 8). Figure 9 shows the progress of the nanoparticles as they approach the outlets; particles are colored by their velocities. These results demonstrate that we are able to simulate tens of thousands of particles in complex (and sparse) geometries.

### 4.2. Computational performance

The strengths of the LBM, in regards to clinical simulation, lie in three key areas: pre-processing, parallel efficiency of simulation (to be discussed in detail in the following), and predictability of time-to-solution.

As a contributor to the time-to-solution, the time required to prepare a geometry for simulation must be factored into the cost of a simulation. Generally speaking, traditional CFD relies on an unstructured-mesh generation procedure to produce a discrete representation of a geometry; complex geometries tend to require high levels of user intervention and considerable CPU time to ensure mesh quality. In comparison, preparation of a geometry for simulation by the LBM requires it to be voxelized: a relatively rapid and simple process that requires little to no user interaction, and only a small fraction of the time-to-solution (since only structured grids are produced). As mentioned previously, we make use of Palabos' voxelization procedure. We use a lattice spacing δ_*x*_ = 25 μm to showcase the capabilities of the drug targeting model, but in practice significantly higher resolution may be required to meet stringent clinical and regulatory standards (e.g. decreasing lattice spacing from 25 to 12 μm results in approximately a 9-fold increase in lattice sites); we benefit greatly from the relative simplicity of voxelization in such instances. Furthermore, because the computational intensity of LBM is predictable (i.e. the variance in the wall-clock time to complete a time-step is minimal), the time-to-solution can be estimated with a high degree of certainty.

Since the LBM is highly parallelizable (and because HemeLB boasts good performance characteristics relative to other codes, as reported in Groen et al., 2013), we have been able to successfully simulate systems consisting of over 1.5 × 10^9^ lattice sites on meaningful time-scales (sufficiently long for most of the particles to have evacuated the geometry), i.e. three cardiac cycles in the case of a resting patient with a heart rate of 68 bpm (using 5,600 ranks of Blue Waters, a petascale supercomputer). In the following section, we present a scalability study of HemeLB using a case consisting of 7.77 × 10^8^ lattice sites.

#### 4.2.1. Scalability

We demonstrate that our memory-optimized version of HemeLB is capable of efficiently simulating large problems on hundreds of thousands of cores, highlighting its potential on petaflops (and beyond) computers; the large-scale simulation of the human arterial tree requires such performance (Grinberg et al., 2009). Our efforts to reduce the memory footprint of the *Initialize* phase (involves the reading of input files, the decomposition of the domain over multiple ranks, and the creation of large data structures that the *Simulate* phase operates on) have allowed for the simulation of flow problems consisting of O(10^9^) lattice sites on Blue Waters. Further work is needed to initialize problems with tens of billions of lattice sites.

Strong scalability of HemeLB (without any particles present, since scalability would be strongly affected by the potential load imbalance caused by the varying distribution of particles) was investigated with the coW15 (15 μm resolution) circle of Willis dataset with 7.77 × 10^8^ lattice sites, executed on the ARCHER Cray XC30 system and built using system GCC 5.1.0 compilers.

ARCHER has dual 12-core Intel Xeon E5-2697v2 (Ivy Bridge) 2.7 GHz processors joined by two QPI links, connected via proprietary Cray Aries interconnect in a dragonfly topology. Some compute nodes have 128 GB of shared memory; however, most have only 64 GB. Executions were performed using fully-populated compute nodes, i.e. each node is assigned 24 MPI ranks (one process per core).

**Figure 10:**
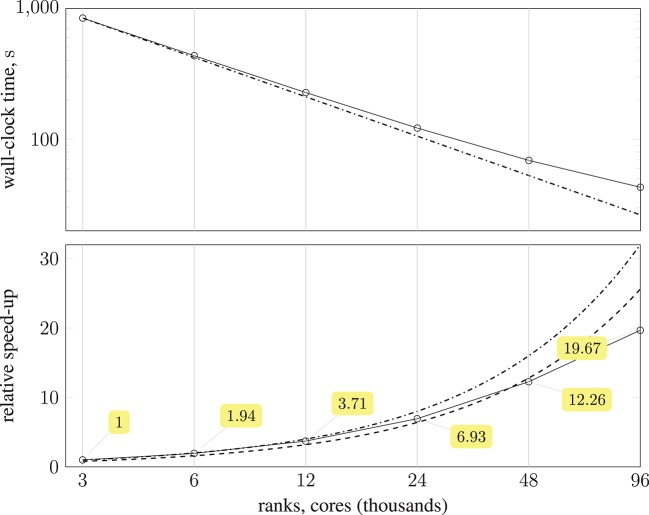
**(Top)** plot shows strong scaling of wall-clock execution time (in seconds) of 10,000 time-steps of *Simulate* phase of HemeLB with coW15 (15 μm resolution) dataset on ARCHER Cray XC30 (24 MPI ranks per compute node) up to 4,000 compute nodes (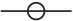); plotted on log-log scale, with dash-dotted line (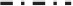) representing perfect scaling. **(Bottom)** plot presents speed-up of HemeLB *Simulate* phase on ARCHER Cray XC30 (24 ranks per node) compared to base configuration using 125 compute nodes (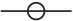). Again, dash-dotted line (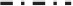) represents perfect scaling and dashed line (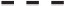) is 80 % of perfect.

The substantial memory requirements of HemeLB with the coW15 test case meant that the smallest configuration required 125 compute nodes (3,000 MPI processes), and progressively larger configurations were run with up to 4,000 compute nodes (96,000 MPI processes). Ten thousand simulation time-steps were executed with periodic writing of the simulation data disabled to reduce variability. The simulation wall-clock execution time and speed-up relative to the smallest execution configuration are shown in Figure 10. Almost a 20-fold speed-up is obtained using 4,000 compute nodes, with 80 % parallel efficiency up to 2,000 compute nodes. Note, by exploiting Streaming SIMD Extensions (SSE), which HemeLB fully supports, we observe a significant ~15% reduction in simulation time.

Performance auditing of HemeLB was done with the open-source Scalasca tool-set (Geimer et al., 2010) for scalable performance analysis of large-scale parallel application executions. Scalasca 2.3.1 with the community-developed Score-P 3.1 instrumentation and measurement infrastructure was used on ARCHER. An instrumented version of HemeLB was prepared with only the main application program and SimulationMaster class selectively instrumented by the GCC compiler, and combined with MPI library interposition. Profiles generated from measured executions were post-processed to derive additional metrics and interactively examined using the Scalasca analysis report explorer.

While the *Initialize* phase of a simulation (when simulation configuration and domain decomposition occurs) requires a roughly constant time to load and distribute the dataset, our primary focus is on the *Simulate* phase (when time-stepping is performed) with its 10,000 time-steps. Also MPI rank 0, which monitors the execution and does not process any part of the simulation data, could be excluded.

**Figure 11:**
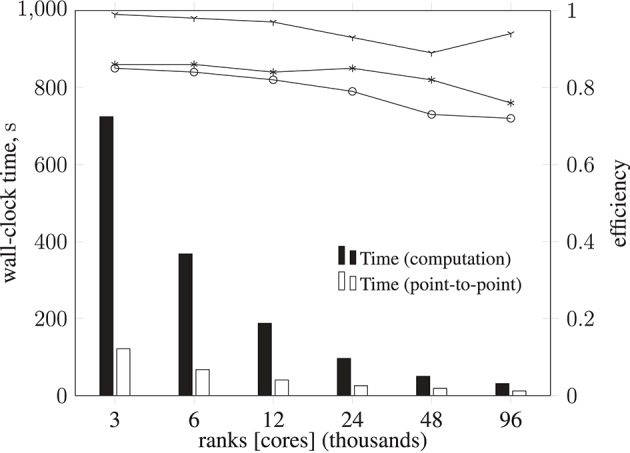
Breakdown of metrics and efficiencies for HemeLB *Simulate* phase (operating on a voxelized representation of the circle of Willis model previously described) on ARCHER Cray XC30 (24 ranks per node). Bars represent, in seconds, the collective communication time, point-to-point communication time, and computation time. Note that the time required for collective operations is negligible; for this reason, the data is not presented. Lines represent communication efficiency (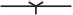), load balance efficiency (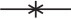), and parallel efficiency (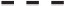). The proportion of computation vs. MPI communication time remains roughly the same (with primarily point-to-point communication and negligible collective communication), with communication efficiency remaining above 0.89. Load balance efficiency starts at 0.86 and progressively deteriorates to 0.76, such that the overall parallel efficiency degrades to 0.72 using 96,000 cores.

A breakdown of the *Simulate* phase CPU time for each execution configuration is shown in Figure 11, along with associated efficiencies. There is a negligible amount of MPI collective communication, and the amount of non-blocking point-to-point communication for data exchange decreases in proportion to computation time. Therefore communication efficiency remains above 0.89. Load balance, however, starts at 0.86 and progressively deteriorates to 0.76, such that the overall parallel efficiency degrades to 0.72 using 96,000 cores. This computational load imbalance will be addressed in future optimization work.

#### 4.2.2. Load balance

**Figure 12:**
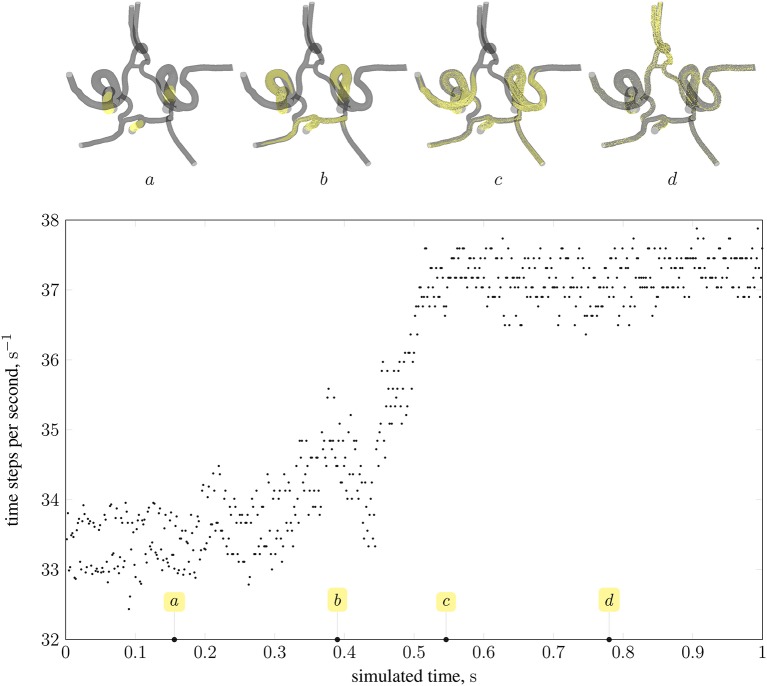
Performance of magnetic drug targeting simulation (all body forces listed in section 2.2.1, including those derived from dipolar interactions, are in effect) in the circle of Willis measured in time-steps per second of wall-clock time (neglecting output steps for clarity). Simulation frames (top) illustrate the particle distribution at four time points in the simulation, indicated on the plot by the tags *a*, *b*, *c*, and *d*.

As stated in the previous section, the distribution of particles affects the load balance. Here, we analyse the imbalance during various stages of a full-scale simulation with δ_*x*_ = 25 μm on 350 nodes (5,600 cores) of Blue Waters, a petascale supercomputer at the National Centre for Supercomputing Applications (NCSA). Figure 12 presents the performance of HemeLB under a simulation of 73,215 nanoparticles injected through the left and right internal carotid arteries, and the basilar artery (all three inlets to the circle of Willis, as shown in Figure 2). Load imbalance due to the accumulation of particles on few ranks (as seen in frame *a* of the figure) results in an average of 33.4 time-steps per second. As the simulation progresses, and particles become more uniformly distributed across ranks (as seen in frames *c* and *d*), the code achieves approximately 37.5 time-steps per second. The same system containing no particles runs at an average of 39 time-steps per second. For comparison, from Figure 10 the code is capable of 23 time-steps per second when δ_*x*_ = 15 μm (and no particles are present) on 250 nodes (6,000 cores) of ARCHER; on 96,000 cores, we compute 232 time-steps per second. Note that because no particles are present in the system, there is no overhead associated with file output. Therefore, in the case presented, the performance degradation is, even in the worst case of load imbalance (33.4 steps per second), not particularly severe.

## 5. Discussion

The application of our magnetic drug targeting model to a patient-specific geometry has allowed us to explore the relevance of various physical properties and design parameters to the manipulation of paramagnetic iron oxide nanoparticles in cerebral blood flow. The physiological environment (e.g. flow and heart rate) determines which forces dominate, and hence the optimum choice of particle properties and magnetic field configuration will vary between patients and target site location. Our computational model intends to facilitate the optimization of these properties for a particular patient, or to predict the percentage of injected particles that will reach a given target site under a fixed configuration (thus potentially advising on the most appropriate dosage or carrier type for that patient).

**Figure 13:**
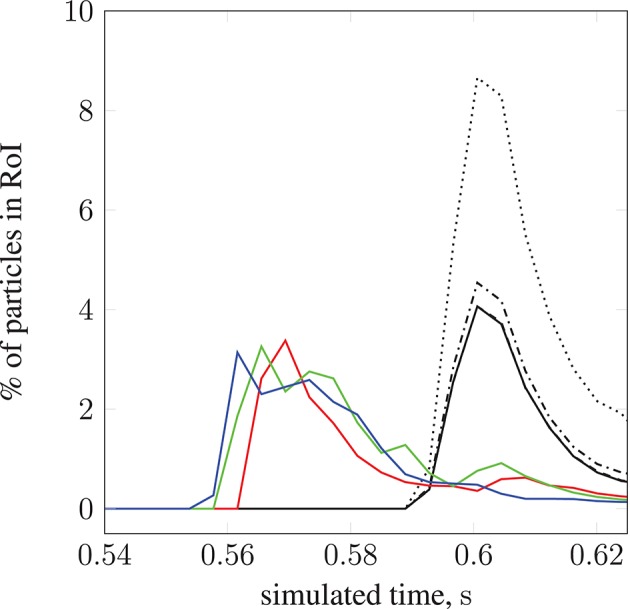
The percentage of particles found in the region of interest (see Figure 7) during simulation. We present results for 2 studies; particle radius *a* is varied in the first, and set to 65 nm (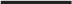), 105 nm (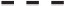), 250 nm (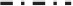), and 500 nm (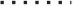). For the second study, particle radius *a* = 65 nm is constant, and the inlet velocity is varied: 10.7 l min^−1^ at 113 bpm (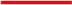), 11.9 l min^−1^ at 120 bpm (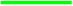), and 13.2 l min^−1^ at 134 bpm (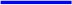).The position of the permanent magnet does not change between cases (held at 0.9 cm from the geometric center of the RoI). A negligible difference is seen when increasing *a* from 65 to 105 nm.

We demonstrate the use of our model with a test case: modeling magnetically steered nanoparticles in a human circle of Willis, with the target site (referred to as the region of interest, RoI) located on a bend in the left internal carotid artery (inlet 2 in Figure 2); an (invasive) magnet placed 0.9 cm from the geometric center of the RoI is used to steer the particles. We study the effects of particle radius on targeting efficiency at the RoI. Figure 7 shows the trajectory of 17,077 particles in the LICA under the influence of a point dipolar magnet of moment ***m***_0_ = {3000.0, 0.0, 0.0}A m^2^. Figure 13 shows the percentage of particles (of radius *a* = 65, 105, 250, and 500 nm) passing through the target region. In physical terms, we find the behavior of the particles to be largely governed by hydrodynamic and dipolar interactions with little contribution from diffusive effects, most likely due to the high flow rates in the given arterial section (~0.8 m s^−1^ peak velocity), which requires a strong magnetic field gradient to overcome drag.

To provide additional insight into the optimization of the particles, we investigate the effect of coating thickness. In the context of drug delivery, for example, the (organic or inorganic) coating surrounding the magnetic core is loaded with the drug. Our implementation of the model can accept a coating thickness *a*_*c*_ (previously assumed to be zero). The application of a coating only affects the drag experienced by the particle, and is assumed to have a negligible effect on the magnetic forcing (i.e. provides no magnetic shielding). With the core radius (*a* =)65 nm, which is used in all calculations pertaining to the magnetic forcing, three coating thicknesses are considered: *a*_*c*_ = 16.25, 32.5, and 65 nm. For the configurations considered, our simulations suggest that particle motion is unaffected by the additional drag due to the coating. On inspection of Equation (8), it is clear that if the local fluid velocity ***v***(*x*_*p*_) at the particle's location *x*_*p*_ is much greater than the velocity modification resulting from any external forcing, i.e.

(13) $$v(x_{p})\gg \beta (F+F_{\text{R}})$$

then any realistic coating will have little influence (since only the mobility β = 1/[6πμ(*a*+*a*_*c*_)] is modified). Because the magnetic field can only (strongly) influence particles within the proximity of the magnet (it falls off as 1/*r*^3^), the current configuration is such that no discernible difference is seen.

**Figure 14:**
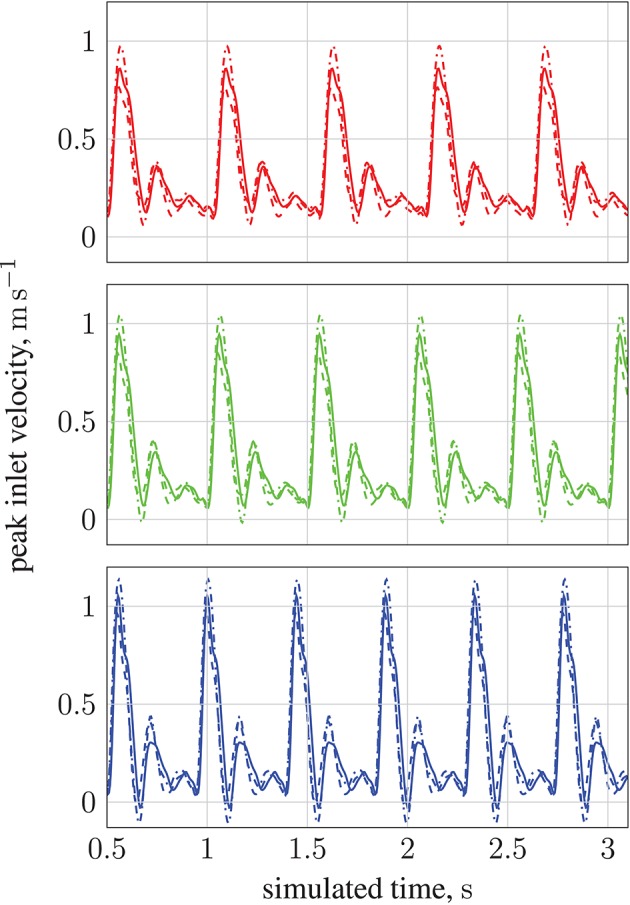
Peak inlet velocity for (1) the basilar artery (*---*), (2) the left internal carotid artery (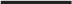), and (3) the right internal carotid artery (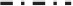). For each of the three inlets, the complete inlet-velocity profile is obtained by assigning weighting factors (of the peak velocity) to lattice sites that lie on the boundaries. The top plot (red) is for 10.7 l min^−1^ at 113 bpm (red line in Figure 13), the middle plot (green) is for 11.9 l min^−1^ at 120 bpm (green line in Figure 13), and the bottom plot (blue) is for 13.2 l min^−1^ at 134 bpm (blue line in Figure 13).

By modifying the velocity profiles of the inlet boundary conditions, we are able to study the impact of three physiological parameters (mean blood pressure, volumetric flow rate, and heart rate at the opening) on particle behavior, demonstrating that our model can handle patient specificity (down to a patient's current physiological state). As a function of these parameters, the values for which we take from the experimental work of Sugawara et al. (2003), the peak inlet velocity is obtained from 1D Navier-Stokes simulations using our multiscale framework (Itani et al., 2015), and introduced to the 3D solver (HemeLB) as scaled parabolic profiles. All simulations presented to this point use the heart rate of a resting patient (80 mmHg, 4.8 l min^−1^, 68 bpm; see Figure 3) to derive inlet boundary conditions. Here, we consider three other cases with greater heart rates (see Figure 14): 112 mmHg, 10.7 l min^−1^, 113 bpm (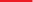); 116 mmHg, 11.9 l min^−1^, 120 bpm (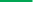); 122 mmHg, 13.2 l min^−1^, 134 bpm (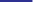). For a fixed particle radius *a* = 65 nm, Figure 13 shows how particle concentration in the RoI is affected. Relative to the case of resting heart rate (—), we see fewer particles in the RoI for higher-flow-rate cases. This is an unsurprising result; as discussed, the relative contribution of magnetic forcing to particle motion is reduced when the fluid velocity is increased. The reduced arrival time of the particles at the RoI is simply due to the greater fluid velocity.

The central parameter controlling hydrodynamic interactions, mediated by frictional coupling, is the particle radius. For particle radius *a* in the range of 65 to 500 nm thermal diffusivity was observed to be negligible. However, diffusive terms introduced by the interaction of the particles with blood cells may well play a significant role. Our current model does not include blood cells in the suspension, but can take into consideration the bulk shear thinning effect resulting from the presence of blood cells; a comparison of Newtonian and non-Newtonian blood models has shown little observable difference in mass flow (Bernabeu et al., 2013).

The effect of gravity (and other homogeneous accelerations) was modeled via a body force term. Our evaluations have found contributions of gravity (buoyancy of the particle, caused by the blood, is also considered) to the dynamics of the particles to be negligible in the test cases presented here. However, with increasing particle size or when considering larger capillary numbers gravity may become significant.

The magnetic properties of the paramagnetic particles are largely determined by the size and crystallinity of their magnetite (or maghemite) core. For simplicity, in the above simulations we have chosen to model particles of pure magnetite. In reality, the magnetite content is expected to be lower, thus reducing the effective magnetic susceptibility χ_*v*_ of a particle. Volumetric magnetic susceptibility, as reported in the literature, varies widely (i.e. 1.0 to 5.7 m^−3^) with the preparation, means of creation, and grain size of the nanoparticles (Hunt et al., 2013); for greatest effect we have chosen the maximum reported value. The size of the particle itself can also affect the susceptibility, as a finite size effect in small particles (e.g. for particle radius *a* ≲ 25 nm Ulbrich et al., 2016) induces super-paramagnetic behavior which manifests as a vastly increased magnetic susceptibility (relative to that of paramagnets). With *a* > 65 nm in the simulations presented here, we neglect to consider the super-paramagnetic regime. Note that our model is able to capture super-paramagnetic behavior, but values for χ_*v*_ would need to be determined experimentally. It is expected that the magnetic susceptibility will be known for any super-paramagnetic iron oxide nanoparticles (SPIONs) used in a clinical context. We have additionally approximated the magnetic permeability inside the brain as that of a classical vacuum, i.e. μ_0_ = 4π × 10^−7^ H m^−1^. In general, the presence of iron rich tissues may cause the magnetic permeability of the surrounding brain matter to deviate from this value.

The initial distribution of the particles, and the invasive proximity of the magnet (both indicated in Figure 7), are clearly unrealistic, and were chosen for illustrative and performance testing purposes. Furthermore, the (single) permanent magnet here is modeled as a pure point dipole, effectively overestimating the field gradient. In future work, particles will be introduced via a timed release at inlets in a manner more closely modeling the concentration profile of an intravenous delivery. In addition, future implementations will model particle function in the target region (such as the absorption of particles into target tissue, or magnetically induced heating of nanoparticles and subsequent drug release). Furthermore, an external electromagnetic field solver will be used to recreate a complex and realistic field (such as may be induced in a clinical context). As stated previously, the input flow velocities for each inlet were obtained using a multiscale approach (to represent the rest of the human arterial tree, Itani et al., 2015), whereas we may wish to consider that in an unhealthy patient the blood pressure and flow rates may be much higher.

Segmentation of the clinical images necessary to construct the three-dimensional vascular geometry is in practice difficult to automate consistently, often needing human intervention to identify artifacts to be filtered out. As a result of this, and other uncertainties in the input data, a number of replica simulations may be required to capture the full statistics of the system, and allow uncertainty quantification of the results. Computational efficiency is therefore very important to the practicality of this model. Currently, the most significant influence on computational performance comes via the distribution of particles across computational subdomains, with large numbers of particles on any single computational subdomain causing load imbalance. While the dilute requirement of our model largely mitigates the problem in high performance computing environments (where core counts of high scaling codes can be increased with relative ease), the transition to smaller workstations using accelerators may require the implementation of sophisticated load balancing techniques. Nevertheless, in the most extreme case of imbalance observed in our simulations, using 5,600 cores (350 nodes) on Blue Waters, the performance was degraded by around ~15% relative to the case where no particles are present—a manageable reduction in performance that can be alleviated through further development of the load balancing techniques employed. To simulate 20,377 particles over three cardiac cycles and with lattice spacing δ_*x*_ = 25 μm using 5,820 cores (220 nodes) on ARCHER requires 20 wall-clock hours. Therefore, based on the scalability study presented in section 4.2.1, and the encouraging results of the load-balance testing involving 73,215 particles, we postulate that our method can simulate tens of thousands of particles over multiple cardiac cycles in geometries consisting of O(10^9^) lattice sites in approximately a day. Such performance allows us to address flow problems that previously could not be approached, and will lead to new a level of understanding.

In order to achieve the necessary computational performance, a number of approximations were implemented. As our particle sizes are significantly smaller than the scale of the lattice discretization (1/25th in the case of the largest particle radius), and with sufficiently low particle density (1–5 particles per lattice volume), we permit ourselves the use of a one-way coupling strategy (no feedback from particles to fluid). Another consequence of the dilute approximation is the use of the much cheaper pairwise expression for the dipolar force (see Equation 9); in practice this would break down for non-dilute fluids. We also assume that particles align instantaneously with the local magnetic field, as the time scale for rotation is extremely rapid (Ulbrich et al., 2016) (relative to the characteristic time-scale of hydrodynamic processes).

## 6. Conclusion

We present an efficient computational model for simulating magnetic drug targeting in patient specific brain geometries, via the steering of paramagnetic nanoparticles with an external magnetic field. The model couples the dynamics of spherical particles to a lattice-Boltzmann hydrodynamics simulation, taking into account body forces (e.g. gravity), diffusivity, and dipolar interactions. A study of the model's computational performance found favorable results, with a performance drop of ~15% (relative to a simulation of the hydrodynamics alone, i.e. in the absence of any particles) in the most extreme case of load imbalance (all particles clustered in one region). We demonstrated the use of the model to predict the particle density (as a function of time) near a target site for a specific patient circle of Willis vascular system and heart rate, using a single point dipolar magnet. Through a multiscale coupling with a 1D representation of the wider vascular system, we obtained inlet velocity profiles for a patient in a range of physiological states (varying heart rate, cardiac output and mean blood pressure). Initial results allow confidence in the viability of the model to answer a wide range of questions relating to the design and manipulation of iron oxide nanoparticles in a clinical context. Comparison to phantom flow results and medical imaging research will allow further tuning of system parameters to further increase the accuracy of the model. A next step toward using the simulation technique in a more realistic manner will involve coupling of the flow solver to a comprehensive electromagnetic simulation. This will allow for the investigation of particle behavior when exposed to more complex magnetic fields created by a combination of multiple electromagnets.

## Author contributions

AP, RR, and SS: Programming of paramagnetic particle controller; RR, AP, and PC: Conception and design of simulations; AP: Performed simulations showcasing capabilities; RR and AP: Analyzed simulation results; BW: Performance audit; AP, RR, SS, BW, RN, and PC: Drafted, edited, and revised manuscript.

### Conflict of interest statement

The authors declare that the research was conducted in the absence of any commercial or financial relationships that could be construed as a potential conflict of interest.

## References

[B1] Ahlrichs P. Dünweg B. (1999). Simulation of a single polymer chain in solution by combining lattice Boltzmann and molecular dynamics. J. Chem. Phys. 111, 8225–8239.

[B2] Alexiou C. Arnold W. Klein R. J. Parak F. G. Hulin P. Bergemann C.. (2000). Locoregional cancer treatment with magnetic drug targeting. Cancer Res. 60, 6641–6648. Available online at: http://cancerres.aacrjournals.org/content/60/23/6641 11118047

[B3] Bernabeu M. O. Jones M. L. Nielsen J. H. Krüger T. Nash R. W. Groen D.. (2014). Computer simulations reveal complex distribution of haemodynamic forces in a mouse retina model of angiogenesis. J. R. Soc. Interface 11:20140543. 10.1098/rsif.2014.0543 25079871 PMC4233731

[B4] Bernabeu M. O. Nash R. W. Groen D. Carver H. B. Hetherington J. Krüger T.. (2013). Impact of blood rheology on wall shear stress in a model of the middle cerebral artery. Interface Focus 3:20120094. 10.1098/rsfs.2012.0094 24427534 PMC3638489

[B5] Berry C. C. Curtis A. S. G. (2003). Functionalisation of magnetic nanoparticles for applications in biomedicine. J. Phys. D Appl. Phys. 36:R198 10.1088/0022-3727/36/13/203

[B6] Birzer C. H. Kalt P. A. Nathan G. J. (2012). The influences of particle mass loading on mean and instantaneous particle distributions in precessing jet flows. Int. J. Multiph. Flow 41(Suppl. C), 13–22. 10.1016/j.ijmultiphaseflow.2011.11.009

[B7] Boivin M. Simonin O. Squires K. D. (1998). Direct numerical simulation of turbulence modulation by particles in isotropic turbulence. J. Fluid Mech. 375, 235–263.

[B8] Bouzidi M. Firdaouss M. Lallemand P. (2001). Momentum transfer of a Boltzmann-lattice fluid with boundaries. Phys. Fluids 13, 3452–3459. 10.1063/1.1399290

[B9] Champion J. A. Katare Y. K. Mitragotri S. (2007). Making polymeric micro- and nanoparticles of complex shapes. Proc. Natl. Acad. Sci. U.S.A. 104, 11901–11904. 10.1073/pnas.0705326104 17620615 PMC1924596

[B10] Coogan J. S. Humphrey J. D. Figueroa C. A. (2013). Computational simulations of hemodynamic changes within thoracic, coronary, and cerebral arteries following early wall remodeling in response to distal aortic coarctation. Biomech. Model. Mechanobiol. 12, 79–93. 10.1007/s10237-012-0383-x 22415052 PMC3545010

[B11] Du D. Biswal S. L. (2014). Micro-mutual-dipolar model for rapid calculation of forces between paramagnetic colloids. Phys. Rev. E Stat. Nonlin. Soft Matter Phys. 90:033310. 10.1103/PhysRevE.90.033310 25314567

[B12] Eftekhar B. Dadmehr M. Ansari S. Ghodsi M. Nazparvar B. Ketabchi E. (2006). Are the distributions of variations of circle of Willis different in different populations? – results of an anatomical study and review of literature. BMC Neurol. 6:22. 10.1186/1471-2377-6-22 16796761 PMC1543654

[B13] Geimer M. Wolf F. Wylie B. J. N. Ábrahám E. Becker D. Mohr B. (2010). The Scalasca performance toolset architecture. Concurr. Comput. 22, 702–719. 10.1002/cpe.1556

[B14] Goodwin S. Peterson C. Hoh C. Bittner C. (1999). Targeting and retention of magnetic targeted carriers (mtcs) enhancing intra-arterial chemotherapy. J. Magn. Magn. Mater. 194, 132–139.

[B15] Grinberg L. Anor T. Madsen J. R. Yakhot A. Karniadakis G. (2009). Large-scale simulation of the human arterial tree. Clin. Exp. Pharmacol. Physiol. 36, 194–205. 10.1111/j.1440-1681.2008.05010.x 18671721

[B16] Groen D. Hetherington J. Carver H. B. Nash R. W. Bernabeu M. O. Coveney P. V. (2013). Analysing and modelling the performance of the HemeLB lattice-Boltzmann simulation environment. J. Comput. Sci. 4, 412–422. 10.1016/j.jocs.2013.03.002

[B17] Guo Z. Zheng C. Shi B. (2002). An extrapolation method for boundary conditions in lattice Boltzmann method. Phys. Fluids 14, 2007–2010. 10.1063/1.1471914

[B18] Horwitz J. Mani A. (2016). Accurate calculation of Stokes drag for point-particle tracking in two-way coupled flows. J. Comput. Phys. 318, 85–109. 10.1016/j.jcp.2016.04.034

[B19] Hunt C. P. Moskowitz B. M. Banerjee S. K. (2013). Magnetic Properties of Rocks and Minerals. American Geophysical Union, 189–204. 10.1029/RF003p0189

[B20] Itani M. A. Schiller U. D. Schmieschek S. Hetherington J. Bernabeu M. O. Chandrashekar H. (2015). An automated multiscale ensemble simulation approach for vascular blood flow. J. Comput. Sci. 9(Suppl. C), 150–155. 10.1016/j.jocs.2015.04.008

[B21] Junk M. Yang Z. (2005). One-point boundary condition for the lattice Boltzmann method. Phys. Rev. E 72:066701. 10.1103/PhysRevE.72.066701 16486092

[B22] Kandelousi M. S. Ellahi R. (2015). Simulation of ferrofluid flow for magnetic drug targeting using the lattice-Boltzmann method. Z. Naturfors. A 70, 115–124. 10.1515/zna-2014-0258

[B23] Kayembe K. N. Sasahara M. Hazama F. (1984). Cerebral aneurysms and variations in the circle of Willis. Stroke 15, 846–850. 6474536 10.1161/01.str.15.5.846

[B24] Kenjereš S. Righolt B. (2012). Simulations of magnetic capturing of drug carriers in the brain vascular system. Int. J. Heat Fluid Flow 35(Suppl. C), 68–75. 10.1016/j.ijheatfluidflow.2012.03.008

[B25] Ladd A. J. C. (1994). Numerical simulations of particulate suspensions via a discretized Boltzmann equation. Part 1. theoretical foundation. J. Fluid Mech. 271, 285–309.

[B26] Larimi M. Ramiar A. Ranjbar A. (2014). Numerical simulation of magnetic nanoparticles targeting in a bifurcation vessel. J. Magn. Magn. Mater. 362, 58–7. 10.1016/j.jmmm.2014.03.002

[B27] Lockman P. R. Mumper R. J. Khan M. A. Allen D. D. (2002). Nanoparticle technology for drug delivery across the blood-brain barrier. Drug Dev. Ind. Pharm. 28, 1–13. 10.1081/DDC-120001481 11858519

[B28] Lübbe A. S. Alexiou C. Bergemann C. (2001). Clinical applications of magnetic drug targeting. J. Surg. Res. 95, 200–206. 10.1006/jsre.2000.6030 11162046

[B29] Lübbe A. S. Bergemann C. Huhnt W. Fricke T. Riess H. Brock J. W.. (1996a). Preclinical experiences with magnetic drug targeting: Tolerance and efficacy. Cancer Res. 56, 4694–4701. 8840986

[B30] Lübbe A. S. Bergemann C. Riess H. Schriever F. Reichardt P. Possinger K.. (1996b). Clinical experiences with magnetic drug targeting: a phase I study with 4′-epidoxorubicin in 14 patients with advanced solid tumors. Cancer Res. 56, 4686–4693. 8840985

[B31] Manini S. Antiga L. Botti L. Remuzzi A. (2015). pyns: An open-source framework for 0d haemodynamic modelling. Ann. Biomed. Eng. 43, 1461–1473. 10.1007/s10439-014-1234-y 25549775

[B32] Maude A. D. (1961). End effects in a falling-sphere viscometer. Br. J. Appl. Phys. 12:293.

[B33] Mazzeo M. D. Coveney P. V. (2008). HemeLB: a high performance parallel lattice-Boltzmann code for large scale fluid flow in complex geometries. Comput. Phys. Commun. 178, 894–914. 10.1016/j.cpc.2008.02.013

[B34] Nacev A. Komaee A. Sarwar A. Probst R. Kim S. H. Emmert-Buck M. (2012). Towards control of magnetic fluids in patients: Directing therapeutic nanoparticles to disease locations. IEEE Control Syst. 32, 32–74. 10.1109/MCS.2012.2189052

[B35] Nash R. W. Adhikari R. Cates M. E. (2008). Singular forces and pointlike colloids in lattice Boltzmann hydrodynamics. Phys. Rev. E 77:026709. 10.1103/PhysRevE.77.026709 18352150

[B36] Nash R. W. Carver H. B. Bernabeu M. O. Hetherington J. Groen D. Krüger T.. (2014). Choice of boundary condition for lattice-Boltzmann simulation of moderate-Reynolds-number flow in complex domains. Phys. Rev. E 89:023303. 10.1103/PhysRevE.89.023303 25353601

[B37] Nguyen N.-Q. Ladd A. J. (2002). Lubrication corrections for lattice-Boltzmann simulations of particle suspensions. Phys. Rev. E Stat. Nonlin. Soft Matter Phys. 66:046708. 10.1103/PhysRevE.66.046708 12443381

[B38] Pankhurst Q. A. Connolly J. Jones S. K. Dobson J. (2003). Applications of magnetic nanoparticles in biomedicine. J. Phys. D Appl. Phys. 36:R167 10.1088/0022-3727/36/13/201

[B39] Plaks A. Tsukerman I. Friedman G. Yellen B. (2003). Generalized finite-element method for magnetized nanoparticles. IEEE Trans. Magn. 39, 1436–1439.

[B40] Pries A. R. Neuhaus D. Gaehtgens P. (1992). Blood viscosity in tube flow: dependence on diameter and hematocrit. Am. J. Physiol. Heart Circ. Physiol. 263, H1770–H1778. 1481902 10.1152/ajpheart.1992.263.6.H1770

[B41] Qian Y. H. Orszag S. A. (1993). Lattice BGK models for the Navier-Stokes equation: nonlinear deviation in compressible regimes. Europhys. Lett. 21:255.

[B42] Radon P. Löwa N. Gutkelch D. Wiekhorst F. (2017). Design and characterization of a device to quantify the magnetic drug targeting efficiency of magnetic nanoparticles in a tube flow phantom by magnetic particle spectroscopy. J. Magn. Magn. Mater. 427, 175–180. 10.1016/j.jmmm.2016.11.008

[B43] Rukshin I. Mohrenweiser J. Yue P. Afkhami S. (2017). Modeling superparamagnetic particles in blood flow for applications in magnetic drug targeting. Fluids 2:29 10.3390/fluids2020029

[B44] Schleich N. Po C. Jacobs D. Ucakar B. Gallez B. Danhier F.. (2014). Comparison of active, passive and magnetic targeting to tumors of multifunctional paclitaxel/spio-loaded nanoparticles for tumor imaging and therapy. J. Control. Release 194(Suppl. C), 82–91. 10.1016/j.jconrel.2014.07.059 25178270

[B45] Shapiro B. Kulkarni S. Nacev A. Muro S. Stepanov P. Y. Weinberg I. N. (2015). Open challenges in magnetic drug targeting. Wiley Interdisc. Rev. Nanomed. Nanobiotechnol. 7, 446–457. 10.1002/wnan.1311 25377422 PMC4397114

[B46] Sugawara J. Tanabe T. Miyachi M. Yamamoto K. Takahashi K. Iemitsu M.. (2003). Non-invasive assessment of cardiac output during exercise in healthy young humans: comparison between modelflow method and doppler echocardiography method. Acta Physiol. Scand. 179, 361–366. 10.1046/j.0001-6772.2003.01211.x 14656373

[B47] Tartaj P. del Puerto Morales M. Veintemillas-Verdaguer S. González-Carreño T. Serna C. J. (2003). The preparation of magnetic nanoparticles for applications in biomedicine. J. Phys. D Appl. Phys. 36:R182 10.1088/0022-3727/36/13/202

[B48] ten Cate A. Nieuwstad C. H. Derksen J. J. den Akker H. E. A. V. (2002). Particle imaging velocimetry experiments and lattice-Boltzmann simulations on a single sphere settling under gravity. Phys. Fluids 14, 4012–4025. 10.1063/1.1512918

[B49] Tietze R. Lyer S. Dürr S. Alexiou C. (2012). Nanoparticles for cancer therapy using magnetic forces. Nanomedicine 7, 447–457. 10.2217/nnm.12.10 22385201

[B50] Torchilin V. P. (2000). Drug targeting. Eur. J. Pharm. Sci. 11(Suppl. 2), S81–S91. 10.1016/S0928-0987(00)00166-4 11033430

[B51] Ulbrich K. Holá K. Šubr V. Bakandritsos A. Tuček J. Zbořil R. (2016). Targeted drug delivery with polymers and magnetic nanoparticles: Covalent and noncovalent approaches, release control, and clinical studies. Chem. Rev. 116, 5338–5431. 10.1021/acs.chemrev.5b00589 27109701

[B52] Winkler D. A. (2017). Computational modelling of magnetic nanoparticle properties and *in vivo* responses. Curr. Med. Chem. 24, 483–496. 10.2174/0929867323666161018141902 27758713

[B53] Yung K. W. Landecker P. B. Villani D. D. (1998). An analytic solution for the force between two magnetic dipoles. Magn. Electr. Separ. 9, 39–52. 10.1155/1998/79537

